# Effect of Basalt Fiber Content on Mechanical Properties of Lunar Regolith Simulant Geopolymer Under Static Loading

**DOI:** 10.3390/ma18194442

**Published:** 2025-09-23

**Authors:** Jianghuai Zhan, Haolan Yi, Neng Wang, Fei Wang, Shuai Li, Jianmin Hua, Xuanyi Xue

**Affiliations:** 1 School of Civil Engineering, Chongqing University, Chongqing 400045, China; 2 School of Management Science and Real Estate, Chongqing University, Chongqing 400045, China; 3 Department of Civil Engineering, The University of Hong Kong, Pokfulam Road, Hong Kong 999077, China

**Keywords:** lunar regolith simulant, geopolymer, test, compressive strength, flexural strength, stress-strain curve

## Abstract

In-situ lunar construction technology is critical for future lunar base development, and the production of geopolymers from lunar regolith—a novel cementitious material with concrete-like properties—has become a vital approach for achieving in-situ resource utilization. This study systematically investigated the influence of basalt fiber content (0–0.4%) on the mechanical properties of lunar regolith simulant geopolymers by controlling key parameters including curing temperature (20 °C and 80 °C), duration (1 d and 7 d), and alkali activator type (strong alkaline solution: a mixture of sodium hydroxide and sodium silicate, and weak alkaline solution: sodium silicate solution). Through testing of 144 specimens, the results revealed that strong alkali-activated specimens with 0.3% fibers cured at 20 °C for 7 d showed optimal ductility with compressive strength of 2.85 MPa and flexural strength of 0.53 MPa, exhibiting characteristic flat stress-strain curves. Specimens with 0.2% fibers under high-temperature curing at 80 °C for 1 d achieved maximum compressive strength of 44.76 MPa and flexural strength of 1.60 MPa but demonstrated brittle failure behavior. Weak alkali-activated specimens containing 0.1% fibers cured at 80 °C for 7 d attained superior comprehensive performance with peak flexural strength reaching 3.88 MPa, showing excellent fiber-matrix synergy. These findings provide important theoretical foundations for optimizing lunar construction materials through customized fiber reinforcement and curing strategies.

## 1. Introduction

With the rapid development of deep space exploration technology, the Moon has become a crucial staging post for future human deep space exploration. Lunar surface scientific base construction, resource exploitation, and other lunar engineering projects have attracted increasing attention [1]. However, the Moon’s extreme environmental conditions—including drastic day-night temperature variations, high vacuum, intense radiation, low gravity, as well as micrometeorite impacts and moonquake activity—pose significant threats to the long-term stability and reliability of artificial facilities [2]. The traditional approach of transporting construction materials from Earth faces substantial economic and sustainability challenges. Estimates indicate that the cost of delivering just 1 kg of supplies to the Moon exceeds one million US dollars [3]. This practical dilemma has prompted the international aerospace community to focus on in-situ resource utilization (ISRU) technology. The lunar surface is covered with abundant regolith and rocks, materials that not only exist in vast quantities but also possess potential as construction raw materials. Utilizing lunar regolith for in-situ construction could dramatically reduce dependence on Earth-based transportation, representing a critical pathway toward establishing sustainable lunar bases.

However, current research primarily relies on ground-based experimental environments, while the acquisition of real lunar regolith samples remains prohibitively expensive and limited in quantity. This reality has made studies utilizing terrestrial simulant materials a crucial pathway for overcoming technical bottlenecks. By precisely replicating the physicochemical properties of lunar regolith simulants (LRS), researchers were able to systematically investigate lunar construction technologies in laboratory settings. These studies provided a reliable theoretical foundation and technical reserves for the future large-scale construction of lunar bases. Currently, several types of LRS have been developed, including JSC-1A [4], LMS-1 [5], NEU-2 [6], KLS-1 [7], CAS-1 [8], HUST-1 [9], BH-1 [10], and CQU-1 [11]. To facilitate their engineering applications, researchers investigated various forming techniques using these LRS materials, such as alkali-activated solidification [12], binder-based consolidation [13,14], sintering [15,16], 3D printing [17,18], and microbial reinforcement [19]. Zhou et al. [10] prepared an alkaline solution using sodium hydroxide and sodium silicate to alkali-activate and solidify BH-1 LRS. They systematically analyzed the mechanical properties of the resulting geopolymer from both macro- and micro-scale perspectives. In a separate study, Carlos Montes et al. [20] developed a novel geopolymer binder composed of lunar regolith. The LRS geopolymer formed through this binder method exhibited excellent radiation shielding and thermal insulation properties while maintaining satisfactory mechanical performance. Simulation analyses demonstrated that construction materials made with this binder could provide sufficient radiation protection for researchers living and working on the lunar surface. Han et al. [9] investigated vacuum sintering and atmospheric sintering techniques for solidifying LRS, examining both the mechanical properties and apparent characteristics of the sintered geopolymer samples. In parallel research, Zhou et al. [21] conducted systematic studies on 3D printable “ink” materials derived from LRS, establishing a comprehensive quantitative evaluation system for assessing material constructability. Their findings revealed that 3D printing technology was frequently combined with alkali activation and binder-based consolidation methods in practical applications. Liu Hanlong’s team [19] applied this technique to LRS, employing calcium carbonate precipitation technology to reinforce the lunar regolith simulant. The research team conducted unconfined compression tests, shear wave velocity measurements, and a series of microscopic analyses to systematically compare the mechanical properties of LRS after different reinforcement cycles. They successfully fabricated lunar regolith bricks and evaluated the feasibility of using microbially induced carbonate precipitation for lunar construction materials. Considering the complex lunar construction environment, alkali-activated solidification presents significant advantages: it enables cast-in-place construction, requires minimal external additives, allows for material recycling, features short preparation cycles, and produces durable building materials with excellent volume stability [22,23]. These characteristics establish alkali-activated solidification as an ideal and mature LRS construction method. Consequently, this study adopted the alkali-activated solidification approach for fabricating LRS geopolymers.

Under the extreme lunar environment, LRS geopolymer materials face significant challenges, including insufficient ductility and susceptibility to cracking under high-low temperature cycling [24], which severely compromise the durability of lunar base structures. Drawing inspiration from fiber-reinforced concrete technology in terrestrial construction engineering, this study proposed utilizing in-situ lunar basalt resources to develop fiber reinforcement materials. Basalt fibers, produced by melting and drawing natural basalt rock at high temperatures, offer exceptional mechanical properties, including high strength, high modulus, outstanding thermal stability across extreme temperatures, and superior corrosion resistance. These characteristics demonstrate remarkable compatibility with the harsh lunar environmental conditions. The lunar surface contains abundant basalt deposits, providing guaranteed in-situ resources for fiber production. Incorporating basalt fibers into LRS geopolymer matrices effectively enhances the material’s ductility and crack resistance, offering an innovative solution for improving both construction quality and long-term operational reliability of lunar bases. Ductility improvement in LRS geopolymers can significantly increase the durability of lunar surface structures. This approach parallels terrestrial construction practices where fiber reinforcement is routinely added to concrete to enhance tensile strength and cracking resistance. As basalt represents an easily accessible in-situ resource on the Moon, its conversion into fiber reinforcement materials presents a practical method for augmenting the ductility of LRS geopolymers.

Current research on basalt fiber reinforcement for improving construction material performance has primarily focused on concrete and asphalt applications, with limited studies investigating its use in LRS geopolymers. The appropriate incorporation of basalt fibers can significantly enhance the mechanical properties of geopolymers. This study systematically investigated the mechanical performance of basalt fiber-reinforced LRS geopolymers by examining five key variables: (1) strong/weak alkali activation solutions, (2) curing temperature, (3) curing duration, and (4) basalt fiber content. The research objectives were threefold: first, to determine the optimal fiber content under various environmental conditions; second, to elucidate the reinforcement mechanisms of basalt fibers in LRS geopolymers; and third, to develop optimized mix design formulas and processing parameters for fiber-reinforced geopolymer materials suitable for lunar base construction.

## 2. Details on LRS Geopolymer and Test Specimen

### 2.1. Materials

**Figure 1:**
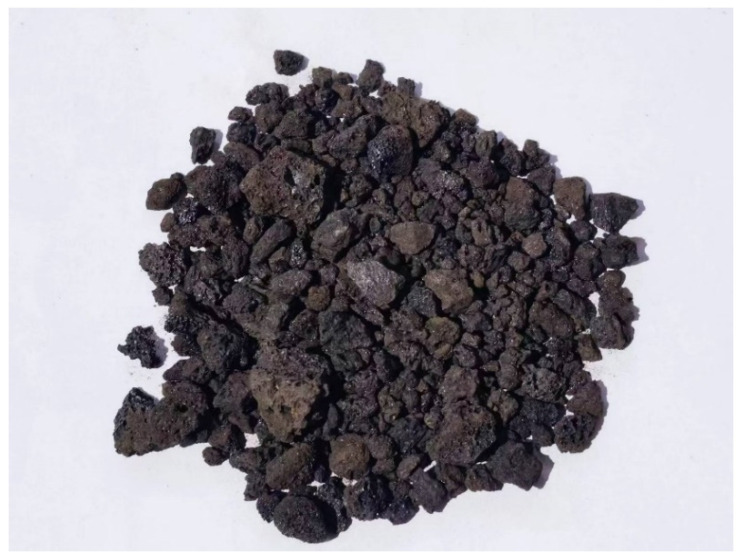
Volcanic slag raw materials.

CQU-1 LRS was prepared using volcanic scoria from Jinlongdingzi volcanic field in Huinan County, Jilin Province, China [25]. The raw volcanic scoria material is shown in Figure 1. Jinlongdingzi volcanic scoria is located at 126°26′ east longitude and 42°20′ north latitude. Its cone primarily consisted of basaltic volcanic scoria, with purplish-red and purplish-gray scoria dominating the upper layers and black scoria mainly distributed in the lower layers. This volcanic ash shared the same origin as other LRS materials, including CAS-1 [8], HUST-1 [9], BH-1 [10], and BH-2 [26]. The material appeared dark brown in color with relatively low hardness. The material exhibited a porous structure with low density, and its chemical composition is presented in Table 1. This study determined the chemical composition of LRS by X-ray fluorescence. The sample was ground to over 200 mesh, dried at 105 °C, mixed with a binder, and pressed into a pellet. Oxide contents were quantified by comparing X-ray intensities against a standard calibration curve. Table 1 compares the chemical composition of CQU-1 LRS with those of authentic lunar regolith and other LRS materials. The results showed that both CQU-1 LRS and real lunar regolith primarily consisted of SiO_2_ and Al_2_O_3_, and exhibited typical aluminosilicate characteristics. The content differences between these major components were negligible. These findings demonstrate that CQU-1 LRS exhibits a chemical composition similar to both lunar regolith and other LRS materials—particularly HUST-1 and BH-1, which share the same raw material origins—confirming its validity as a representative lunar regolith simulant.

**Figure 2:**
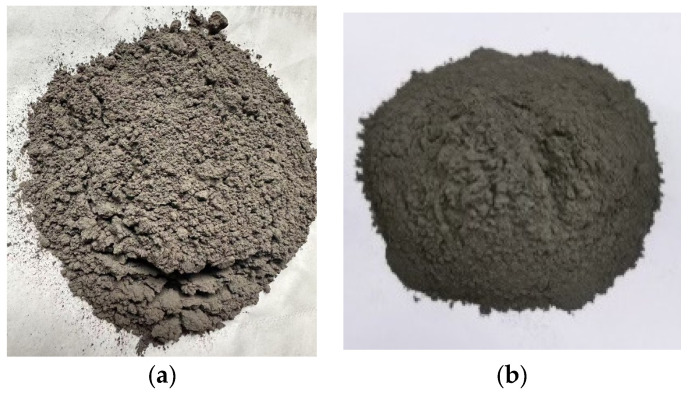
Comparison of lunar regolith simulants (**a**) CQU-1 and (**b**) HUST-1 [9].

**Figure 3:**
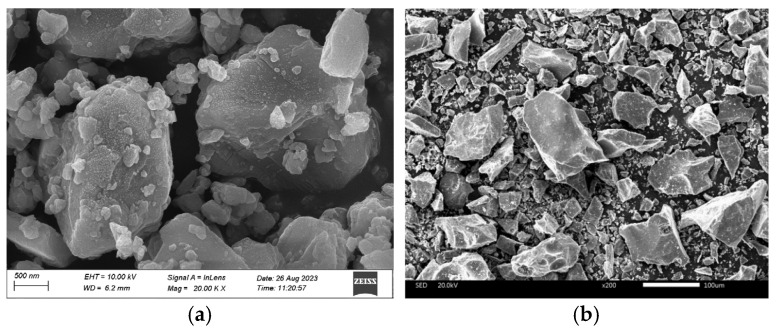
Microscopic comparison of lunar regolith simulants (**a**) CQU-1 and (**b**) HUST-1 [9].

CQU-1 LRS exhibited a powdery morphology in appearance, with its macroscopic characteristics shown in Figure 2. These macroscopic features closely resembled those of HUST-1 LRS [9]. Microscopic comparison (Figure 3) further revealed that the CQU-1 LRS particles displayed angular shapes, which were also similar to the morphological characteristics of HUST-1 LRS.

**Figure 4:**
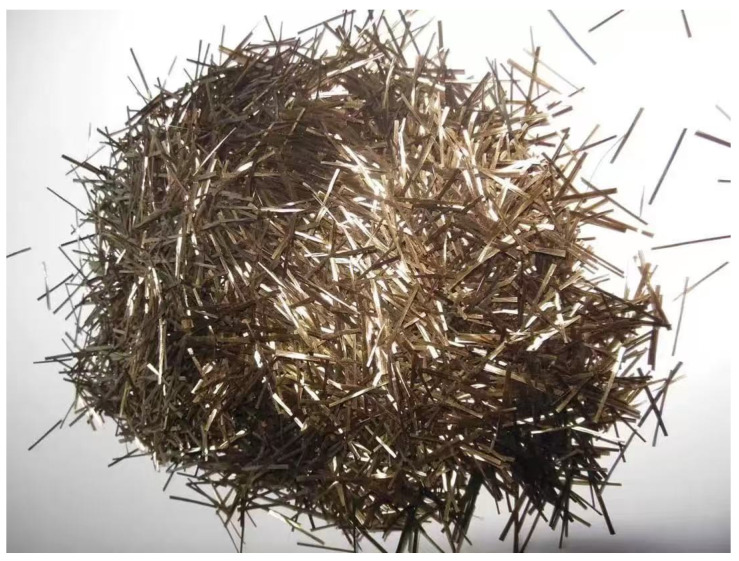
Basalt fiber.

The sodium silicate solution (modulus: 3.3) used in this study was commercially obtained from Shandong Yousu Chemical Technology Co., Ltd. (Linyi, China), with detailed parameters listed in Table 2, while the sodium hydroxide flakes (purity: 99%) were supplied by Ningxia Jinyuyuan Chemical Group Co., Ltd. (Yinchuan, China). The geopolymers were prepared using CQU-1 LRS as the base material. The basalt fibers, produced by China Anjie Company (Jiaxing, China), exhibited the following properties: monofilament diameter of 10 μm, density ranging from 2.63 to 2.65 g/cm^3^, elastic modulus between 91 and 110 GPa, and tensile strength varying from 3000 to 4800 MPa, as fully specified in Table 3. The basalt fibers used in this study had a length of 12 mm, as shown in Figure 4. In this study, the content of basalt fiber is calculated by mass, expressed as the percentage of the mass of basalt fiber relative to the mass of LRS materials (cementitious materials). The volume percentages of basalt fiber corresponding to mass fractions of 0.1%, 0.2%, 0.3%, 0.4%, and 0.5% are 0.077%, 0.154%, 0.231%, 0.308%, and 0.385%, respectively, with an aspect ratio of 12 mm/10 µm = 1200.

### 2.2. Geopolymer Specimen Preparation

The sodium silicate solution contains 26.5% SiO_2_, 8.3% Na_2_O, and 65.2% water by mass. The modulus (Ms) is defined as the molar ratio of SiO_2_ to Na_2_O (Ms = n(SiO_2_)/n(Na_2_O)). The modulus can be adjusted by introducing additional sodium hydroxide to achieve the desired alkali activation environment. Alkali content is expressed as the percentage mass of Na_2_O. The Na_2_O content is calculated as the percentage of the total Na_2_O (from both the additional source and the sodium silicate solution) relative to the mass of LRS raw material.

Since the water content in the sodium silicate solution is fixed, the water-binder ratio (w/b) can be controlled by adjusting the dosages of sodium silicate, LRS raw material, and additional sodium hydroxide. In this study, the strong alkaline solution was prepared by mixing sodium silicate with additional sodium hydroxide, while the weak alkaline solution consisted solely of sodium silicate without any supplementary NaOH. For the strong alkaline solution, the water-binder ratios were prepared according to reference [11], with a fixed w/b of 0.25.

**Figure 5:**
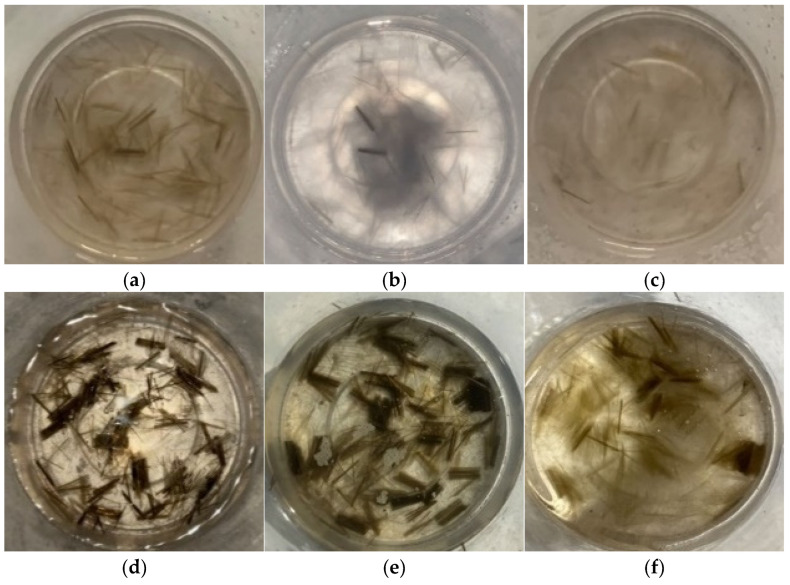
Basalt fibers in strong and weak alkaline solution environments. (**a**) As a cured state under strong alkali activation with standard curing. (**b**) 1 d of standard curing under strong alkali activation. (**c**) 7 d of high-temperature curing at 80 °C under strong alkali activation. (**d**) As a cured state under weak alkali activation with standard curing. (**e**) 1 d of standard curing under weak alkali activation. (**f**) 7 d of high-temperature curing at 80 °C under weak alkali activation.

To ensure clearer experimental results and more rigorous conclusions, we conducted the following two preliminary tests before finalizing the alkali solution type and water-binder ratio for the weak alkali solution:

(1) Stability of basalt fibers in strong/weak alkali environments. As shown in Figure 5, under standard curing conditions (20 ± 2 °C, relative humidity ≥ 95%; controlled at 20 °C in this study), basalt fibers showed negligible reaction with strong alkali solutions. However, in high-temperature environments (80 °C), the strong alkali solution caused visible corrosion of the fibers, resulting in blurred contours. In contrast, at 80 °C, basalt fibers reacted only partially with the weak alkali solution, and the mixture exhibited a gel-like state after high-temperature curing.

(2) In determining the water-binder ratio for the weak alkali solution, this study referenced Debbarma’s research [12], when using pure sodium silicate solution for alkali activation, weak alkaline solutions with water-binder ratios of 0.456 and 0.326 were adopted to prepare geopolymers through flexural tests, aiming to explore the optimal water-binder ratio for weak alkali activation of CQU-1 LRS geopolymer. The results revealed that when the water-binder ratio of the weak alkaline solution was 0.456, the geopolymer exhibited superior ultimate flexural strength and ultimate strain.

During the geopolymer preparation process, the paste demonstrated poor fluidity at a water-binder ratio of 0.326, failing to flow spontaneously and requiring external force for compaction. In contrast, at a water-binder ratio of 0.456, the paste exhibited satisfactory fluidity, enabling self-flow and facilitating vibration-assisted molding. Consequently, a water-binder ratio of 0.456 was selected for the weak alkaline solution in this study.

In this study, strong and weak alkali solutions were employed as alkali activators, with their specific formulation parameters detailed in Table 4. The strong alkali solution was prepared by blending sodium silicate solution with sodium hydroxide, exhibiting a modulus of 1.5 and an alkali content of 10. Geopolymer samples were fabricated using this solution at a water-binder ratio of 0.25. Conversely, the weak alkali solution consisted solely of sodium silicate solution with a modulus of 3.3 and an alkali content of 8.3, which was used to prepare geopolymers at a water-binder ratio of 0.456. This comparative parameter design enabled systematic investigation of the effects of different alkaline environments on geopolymer performance.

This study investigated the relationship between early-age strength development and curing temperature for LRS geopolymers, building upon existing literature [29]. Two curing regimes were employed: standard curing at 20 °C and high-temperature curing at 80 °C. Following the Chinese national standard “Standard for Test Methods of Concrete Physical and Mechanical Properties” (GB/T 50081-2019) [30], mechanical properties were evaluated at two curing ages (1 d and 7 d) to systematically examine the influence of curing duration on the material’s early-age strength development.

**Figure 6:**
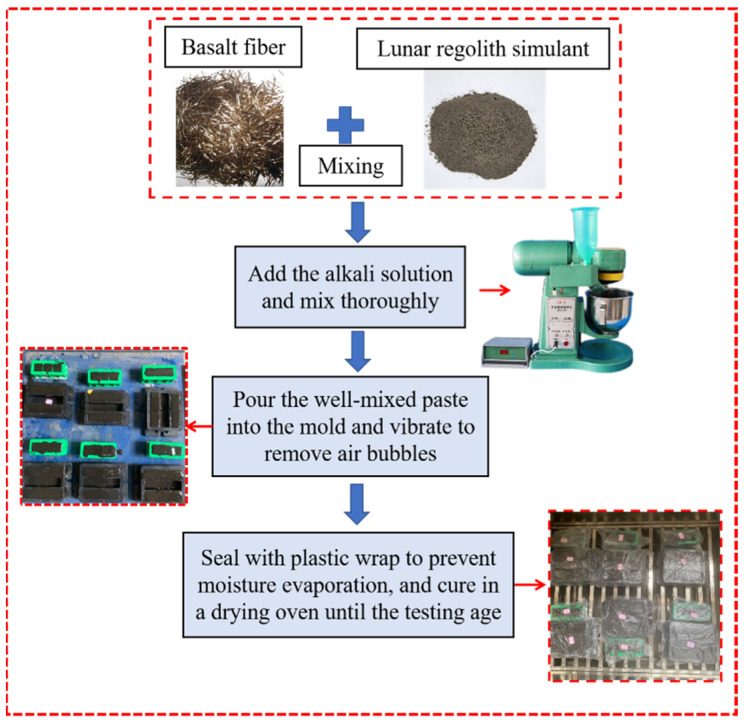
Test procedure.

The experimental procedure began with the preparation of a strong alkaline solution (10% alkali content, modulus 1.5) 24 h in advance by mixing sodium hydroxide, sodium silicate, and deionized water to prevent interference from exothermic reactions during immediate preparation, while petroleum jelly was applied to mold surfaces to facilitate demolding. The CQU-1 LRS was then mixed with basalt fibers at 140 ± 5 r/min to prevent agglomeration before adding the alkaline solution, followed by sequential mixing at 140 ± 5 r/min for 2 min and 285 ± 10 r/min for 3 min to obtain a homogeneous geopolymer paste. According to GB/T 17671-2021 [31], the paste was poured into 40 mm cube and 40 × 40 × 160 mm prism molds, compacted on a vibrating table to remove air bubbles, leveled and sealed with polyethylene film for curing at either 20 °C or 80 °C, with long-term specimens being demolded after 24 h, wrapped in film and further cured in a drying oven to specified ages, as illustrated in the complete workflow (Figure 6). The mixed proportions of the experiments are presented in Table 5.

Based on the preliminary experimental results, no significant reaction was observed between the basalt fibers and the strong alkali solution under standard curing conditions. Therefore, the strong alkali solution was selected as the alkali activator to investigate the effects of standard curing (20 ± 2 °C, relative humidity ≥ 95%) on the early-age strength development of LRS geopolymers.

**Figure 7:**
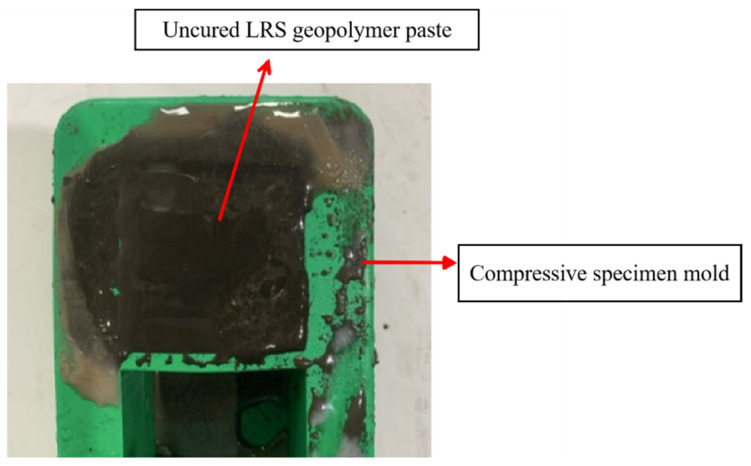
LRS geopolymers cured by strong alkali at 20 °C room temperature for 1 d.

After casting the LRS geopolymer paste into molds following standard procedures and curing at 20 °C for 24 h, the forming condition is shown in Figure 7. The cured specimens failed to achieve hardening, maintaining a paste-like state with measurable fluidity both externally and internally. Testing revealed that minor external forces caused immediate deformation, rendering demolding impossible and demonstrating negligible load-bearing capacity. These results clearly indicated that strong alkali-activated LRS geopolymers could not achieve proper formation under 20 °C/24 h curing conditions. Consequently, only three curing regimes were adopted for subsequent experimental analysis: (1) strong alkali activation with 7 d of standard curing at 20 °C, (2) strong alkali activation with 1 d of high-temperature curing at 80 °C, and (3) weak alkali activation with 7 d of high-temperature curing at 80 °C. This selection was based on a comprehensive evaluation of workability, demolding feasibility, and preliminary strength development characteristics.

## 3. Experimental Methods

**Figure 8:**
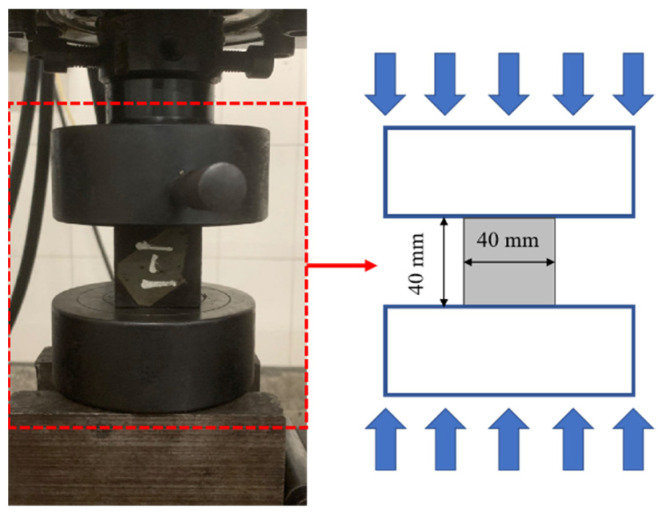
Compressive strength test.

Uniaxial compression tests were conducted using a TSE105D microcomputer-controlled electronic universal testing machine, as shown in Figure 8. Three parallel specimens were tested for each variable design, totaling 72 specimens for compressive tests and 72 specimens for flexural tests, with a grand total of 144 specimens. The loading process was displacement-controlled at a rate of 0.1 mm/min. During testing, load data, axial compressive deformation, and stress-strain curves were recorded to calculate the elastic modulus of basalt fiber-reinforced LRS geopolymer specimens. The loading was terminated when the specimen fractured or when the load exhibited a significant reduction. The compressive strength was calculated according to Equation (1), as follows:

(1) $$R_{c}=\frac{F_{c}}{A}$$

where *R_c_* represents the compressive strength in megapascals (MPa), *F_c_* denotes the maximum load at failure in newtons (N), and *A* is the cross-sectional bearing area in square millimeters (mm^2^).

**Figure 9:**
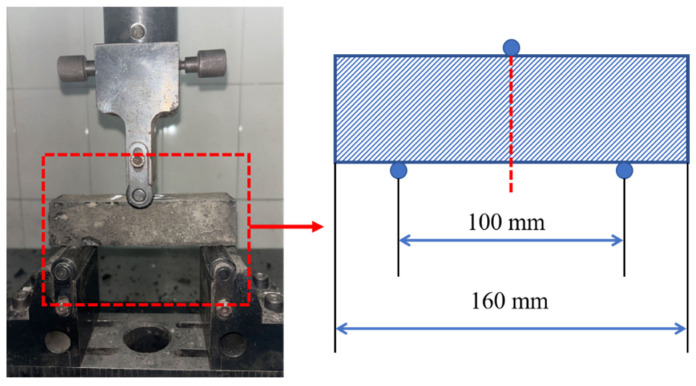
Flexural strength test.

The flexural strength was determined using the three-point bending method, as illustrated in Figure 9. By adjusting the loading fixture of the TSE105D microcomputer-controlled electronic universal testing machine, the flexural strength testing was performed. The loading point was positioned at the midspan of the specimen, 50 mm from each of the two supports spaced 100 mm apart, and the loading process was displacement-controlled at a rate of 0.1 mm/min. The test was terminated when a specimen fracture occurred or when the loading force exhibited a significant reduction. Throughout the test, the applied load, axial deformation of the specimen, and failure mode were recorded. The flexural strength was calculated according to Equation (2), which is [31] the following:

(2) $$R_{f}=\frac{1.5F_{f}L}{b^{3}}$$

where *R_f_* represents the flexural strength in megapascals (MPa), *F_f_* denotes the load applied at the midpoint of the prism at fracture in newtons (N), *L* is the span length between supporting rollers in millimeters (mm), and *b* indicates the edge length of the prism’s square cross-section in millimeters (mm).

## 4. Test Results

### 4.1. Stress-Strain Curve

**Figure 10:**
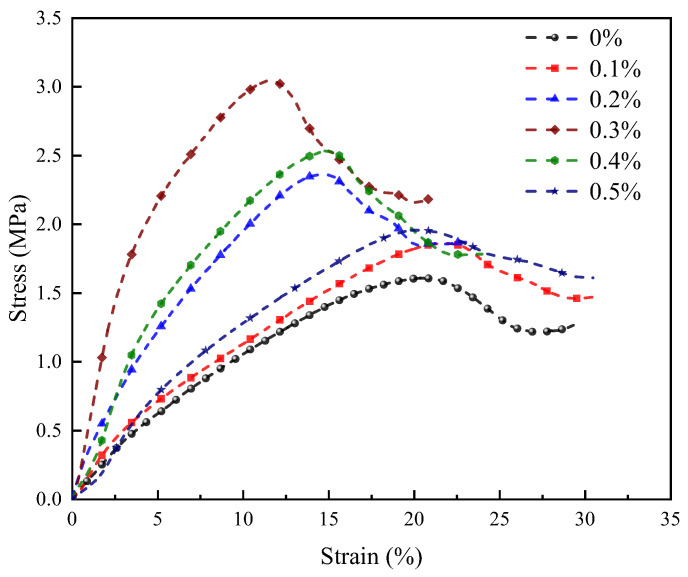
Stress-strain curves of LRS geopolymers with different fiber contents under strong alkali activation and cured at 20 °C for 7 d.

In this study, the stress-strain curves of LRS geopolymer represent the average curves of three specimens. Figure 10 presents the uniaxial compressive stress-strain behavior of alkali-activated LRS geopolymers after 7 d of curing, showing that basalt fiber content significantly influences mechanical properties. Fiber-free specimens displayed brittle fracture with linear-elastic response and sudden failure at peak stress. Basalt fiber incorporation enhanced peak strength, with optimal performance observed at 0.3% fiber content, where curves transitioned from linear-elastic to strain-hardening behavior. This transition featured increased elastic slope, elevated peak stresses at reduced strains, and decreased peak-yield spacing, indicating effective microcrack suppression. Beyond 0.3% fiber content, the behavior reverted toward linear-elastic failure with reduced peak stresses and slower strength development. However, all fiber-reinforced specimens maintained extended peak-yield spacing compared to controls, prolonging failure progression and retaining partial load capacity after cracking. The improved toughness directly resulted from fiber bridging effects that controlled crack propagation. These findings demonstrate the critical role of fiber dispersion in optimizing geopolymer mechanical performance.

**Figure 11:**
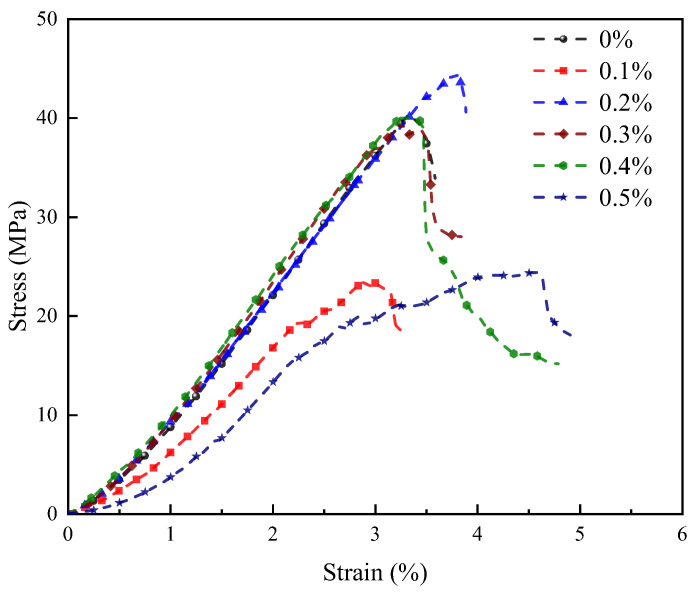
Stress-strain curves of LRS geopolymers with different fiber contents under strong alkali activation and cured at 80 °C for 1 d.

The stress-strain curves of alkali-activated LRS geopolymers after 1 d of curing at 80 °C are shown in Figure 11. High-temperature curing enhanced brittle characteristics compared to standard curing, likely due to accelerated reaction-induced microstructural changes. At 0.4% fiber content, a distinct post-peak plateau emerged, indicating optimal toughening. The 0.5% fiber specimen exhibited linear-elastic failure, suggesting fiber clustering-induced matrix inhomogeneity. Specimens with 0–0.4% fibers displayed strain-hardening behavior with comparable elastic stages. Basalt fibers significantly improved both peak strength and deformation capacity, while appropriate fiber content increased the stress-strain curve area, confirming enhanced composite toughness.

**Figure 12:**
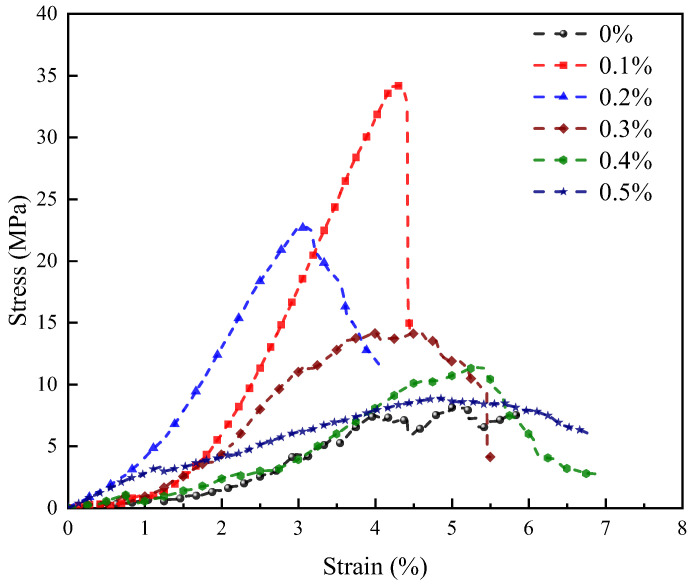
Stress-strain curves of LRS geopolymers with different fiber contents under weak alkali activation and cured at 80 °C for 7 d.

Figure 12 presents the stress-strain curves of weak alkali-activated LRS geopolymers cured at 80 °C for 7 d, demonstrating the critical influence of basalt fiber content on mechanical performance. At 0.1–0.2% fiber content, the curves exhibited strain-hardening behavior, while fiber-free and high-fiber specimens with content exceeding 0.2% displayed linear-elastic responses. The optimal fiber content of 0.1% enhanced peak strength and stiffness but reduced ductility, leading to brittle failure following rapid strength increase. Excessive fiber incorporation increased microporosity, which reduced stiffness while improving ductility through pore-compaction effects.

Nonlinear curve evolution revealed multi-phase reinforcement mechanisms: microcrack initiation dominated low-strain stages, fiber-matrix debonding controlled peak-stress behavior, and fiber bridging influenced post-peak responses. Curing conditions significantly altered these features—standard curing shortened pre-peak nonlinearity, high-temperature curing enhanced post-peak plateaus, and weak-alkali conditions introduced complex pore-compaction effects, reflecting curing-dependent interfacial properties.

### 4.2. Failure Mode Under Static Loading

**Figure 13:**
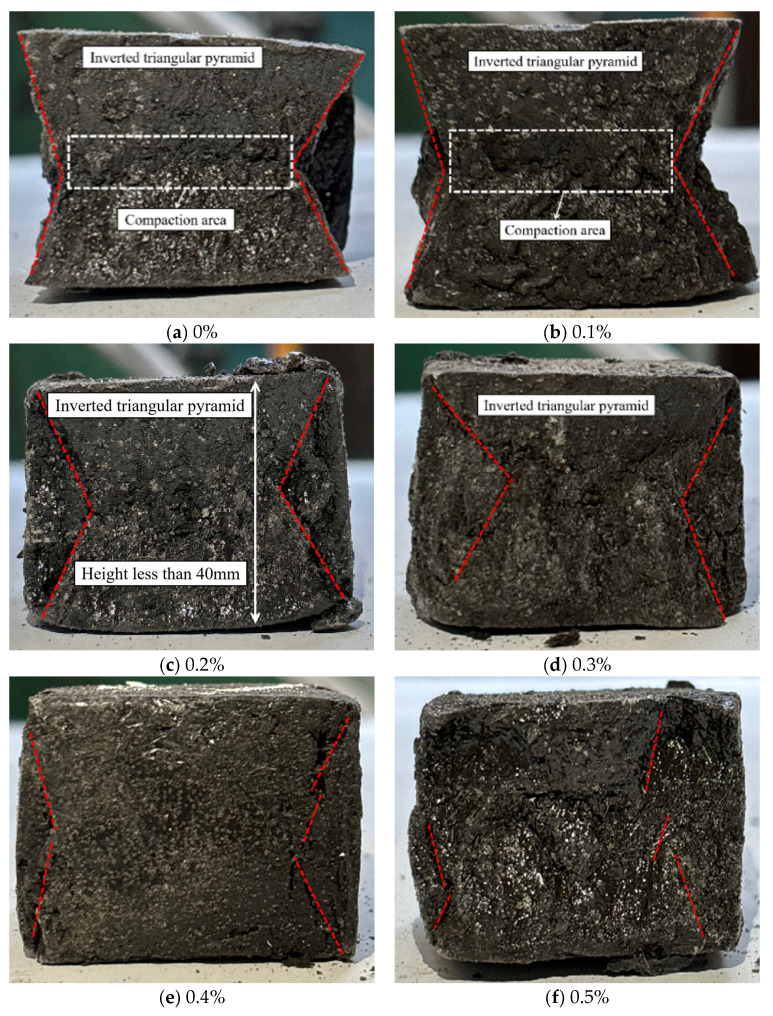
Compressive failure modes of LRS geopolymers with different fiber contents under strong alkali activation and cured at 20 °C for 7 d.

Figure 13 shows the compressive failure modes of alkali-activated LRS geopolymers after 7 d of curing. Specimens displayed ductile failure characteristics, including soft texture, no audible cracking, significant height reduction, lateral expansion, and eventual flattening, consistent with stress-strain behavior. The hoop effect was evident at 0–0.2% fiber content, faded at 0.3%, and vanished at 0.4–0.5%. Peripheral debris spalling occurred at 0–0.2% fiber content but diminished with higher fiber additions.

**Figure 14:**
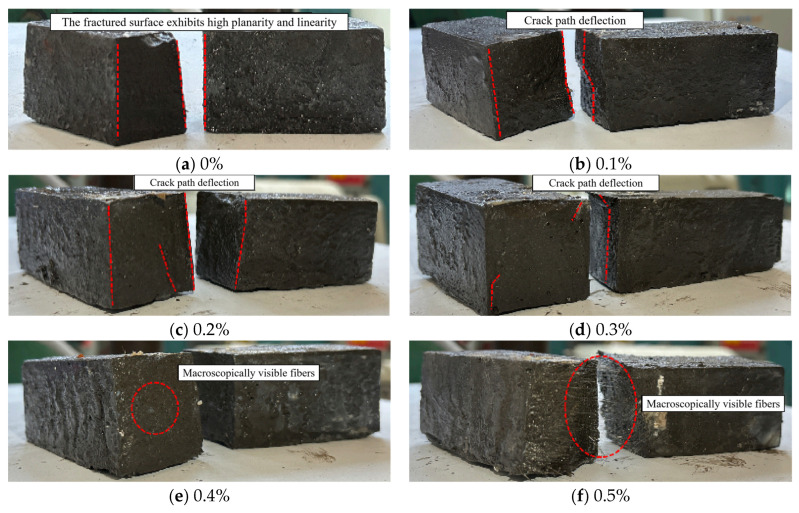
Flexural failure modes of LRS geopolymers with different fiber contents under strong alkali activation and cured at 20 °C for 7 d.

Figure 14 shows the flexural failure modes of alkali-activated LRS geopolymers after 7 d of curing. Fracture surfaces revealed more visible fibers at higher contents, demonstrating basalt fibers’ corrosion resistance in the alkaline environment. All specimens showed ductile failure with progressive crack development. Unreinforced specimens failed with straight fractures and cracks below 0.1 mm in width, while fiber-reinforced specimens exhibited wider cracks measuring 0.2 to 0.5 mm and fracture planes inclined at 30 to 45 degrees, confirming effective fiber bridging. The optimal fiber content of 0.2 percent improved toughness by 40 percent, but higher contents above 0.3 percent caused porosity increases of 15 to 25 percent and reduced interfacial bond strength by 10 to 15 percent due to fiber clustering. These results demonstrate fiber content’s critical role in governing both the macroscopic failure behavior and structural integrity of LRS geopolymers.

**Figure 15:**
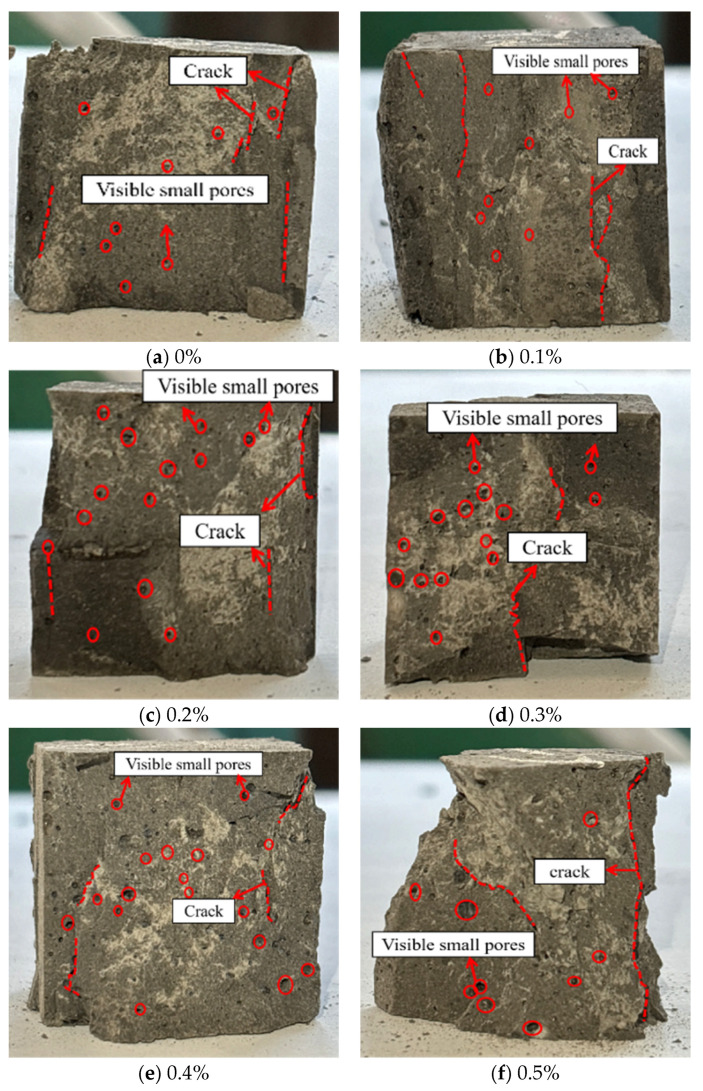
Compressive failure modes of LRS geopolymers with different fiber contents under strong alkali activation and cured at 80 °C for 1 d.

Figure 15 presents the compressive failure modes of LRS geopolymers with varying fiber contents after 1 d of curing at 80 °C under strong alkaline conditions. Uniaxial compression tests at 0.1 mm/min revealed brittle failure characteristics, evidenced by audible cracking sounds and sudden load drops post-peak stress. Increasing basalt fiber content led to greater porosity and larger pores due to reduced workability during casting and fiber corrosion under high-temperature alkaline conditions.

The addition of basalt fibers increased the flexural strength of LRS geopolymers by 11% to 40% compared to the control group. Specifically, 0.1%, 0.2%, 0.3%, 0.4%, and 0.5% fiber content improved strength by 33%, 40%, 20%, 12%, and 11%, respectively. Flexural strength peaked at 1.6 MPa with 0.2% fibers, first rising then declining with higher fiber content. The most rapid increase occurred at 0.0–0.1%, followed by a sharp drop between 0.2–0.3%, then stabilizing. As reported in [12], fiber bridging allowed sustained stress resistance in the tension zone until failure, enhancing flexural strength, which matches this study’s findings.

**Figure 16:**
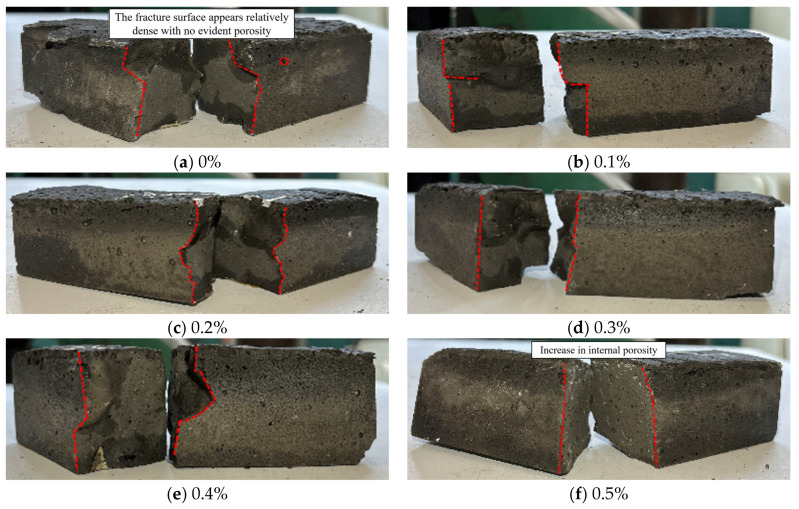
Flexural failure modes of LRS geopolymers with different fiber contents under strong alkali activation and cured at 80 °C for 1 d.

Figure 16 shows the failure modes of LRS geopolymer flexural specimens with different fiber contents under strong alkaline conditions after 1 d of curing at 80 °C. During bending tests, the control specimens failed suddenly. As the load increased, the specimens fractured rapidly into two parts after cracking, showing brittle failure behavior. With fiber addition, the specimens exhibited a delayed period between crack initiation and final fracture under increasing load, failing more slowly than the control group. This was due to the fiber bridging effect that improved the ductility of LRS geopolymers. As fiber content increased, the fracture surfaces showed larger and more numerous pores, corresponding to reduced flexural strength. The main reasons were similar to those for compressive specimens: (1) increased fiber content reduced mixture workability, leaving more pores during casting; (2) under high-temperature, strong alkaline conditions, corrosion of basalt fibers inside the specimens created additional pores, affecting the internal structure.

**Figure 17:**
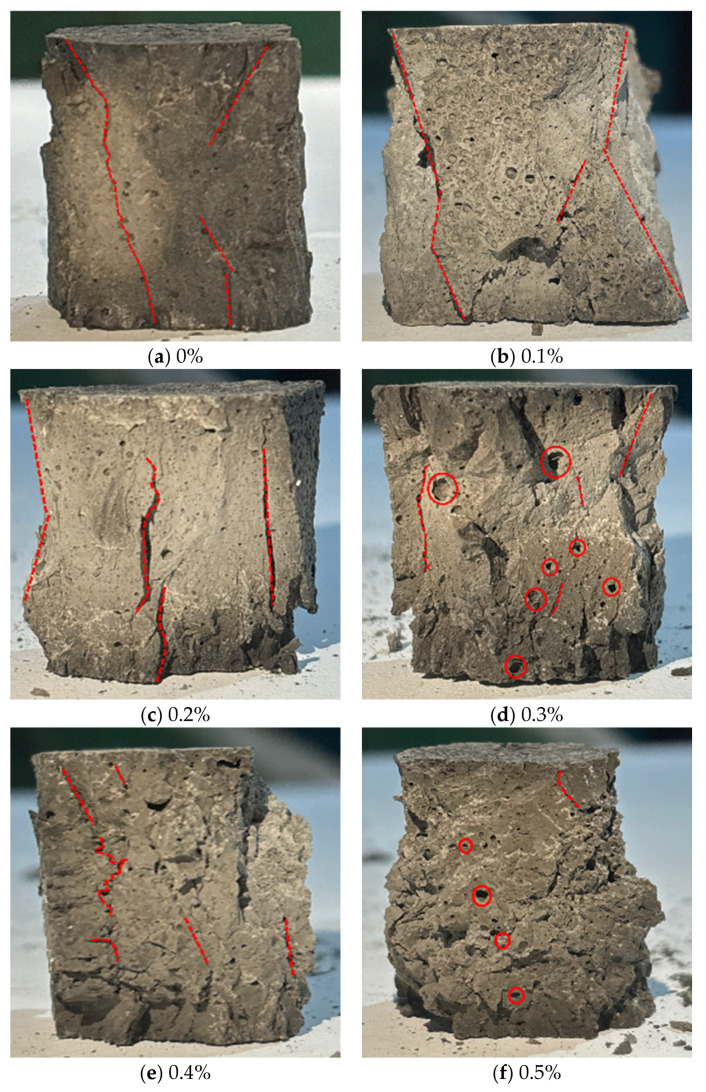
Compressive failure modes of LRS geopolymers with different fiber contents under weak alkali activation and cured at 80 °C for 7 d.

Figure 17 presents the compressive failure modes of LRS geopolymers with varying fiber contents after 7 d of curing in a weak alkaline solution at 80 °C. Control specimens exhibited rapid brittle failure with crisp sounds and debris spalling, assuming pyramid shapes post-failure. Fiber-reinforced specimens demonstrated slower failure progression with duller sounds and peripheral spalling, though maintaining brittle characteristics with outwardly bulging cross-sections.

Under strong alkaline conditions with 80 °C curing, fiber-reinforced specimens consistently showed enhanced flexural strength regardless of fiber content. However, weak alkaline conditions revealed different behavior—excessive fiber content (beyond 0.3%) reduced flexural performance through three mechanisms: increased internal porosity; decreased matrix pH, reducing geopolymeric particle solubility; and compromised mixture fluidity. These effects collectively contributed to flexural strength degradation, as documented in reference [32]. The study highlights how environmental conditions critically influence fiber-reinforced geopolymer performance, with weak alkaline environments being particularly sensitive to fiber dosage.

**Figure 18:**
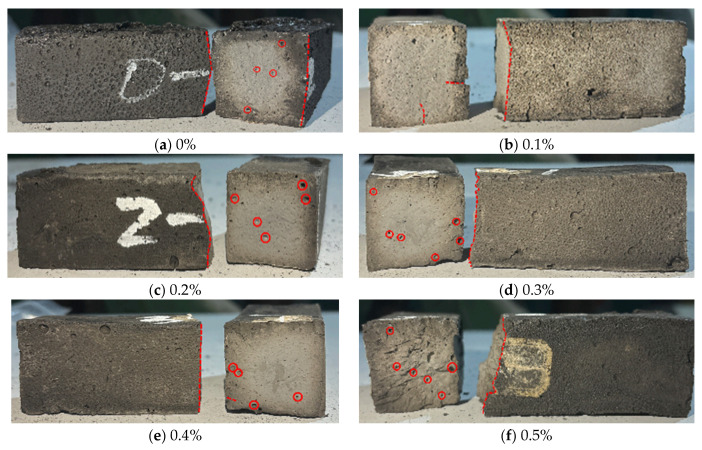
Flexural failure modes of LRS geopolymers with different fiber contents under weak alkali activation and cured at 80 °C for 7 d.

Figure 18 shows the flexural failure modes of LRS geopolymer specimens with varying fiber contents after 7 d of thermal curing at 80 °C in a weak alkaline solution. Control specimens failed abruptly, breaking into two parts upon cracking, displaying brittle behavior. In contrast, fiber-reinforced specimens showed delayed fracture after crack initiation, indicating enhanced ductility. Fracture surfaces exhibited whitish discoloration at their centers, suggesting high-temperature reactions between the alkaline solution, basalt fibers, and LRS, which formed white gel-like polymers. With increasing fiber content, fracture surfaces grew more irregular, implying excessive fibers disrupted internal load-bearing structures, increasing microporosity and reducing flexural strength.

### 4.3. Compressive Strength Under Static Loading

**Figure 19:**
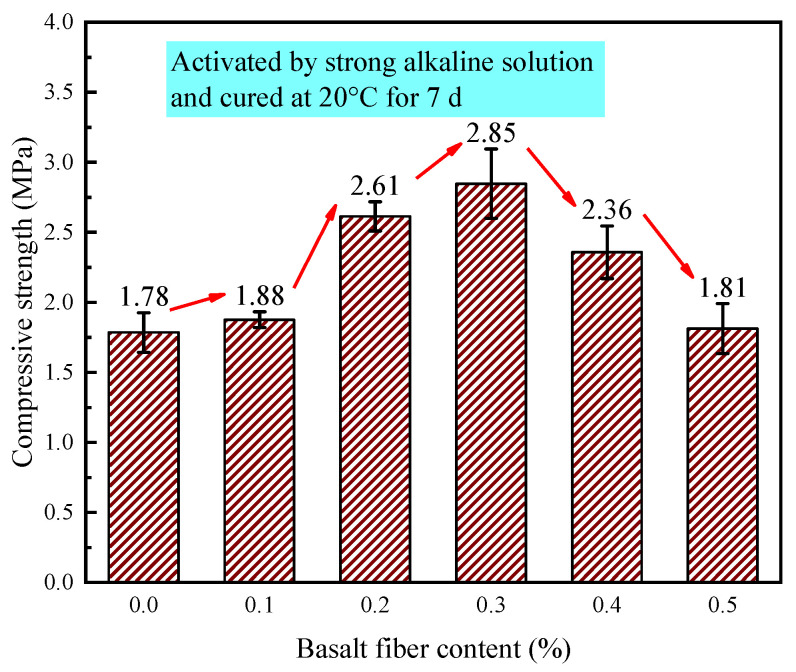
Compressive strength of LRS geopolymers with different fiber contents under strong alkali activation and cured at 20 °C for 7 d.

The effect of varying fiber contents on the compressive strength of LRS geopolymers under strong alkaline environment with standard 7 d curing conditions is presented in Figure 19. The compressive strength of LRS geopolymers ranged between 1.78 and 2.85 MPa. When the basalt fiber content reached 0.3%, the compressive strength peaked, showing a 60.1% improvement compared to the control group. The incorporation of basalt fibers consistently enhanced the compressive strength of LRS geopolymers. As the basalt fiber content increased, the compressive strength initially rose and then declined, a trend consistent with findings reported by Jalasutram et al. [33]. However, when 0.1% and 0.5% basalt fibers were added, the strength improvement was not significant. This phenomenon could be attributed to (1) at 0.1% fiber content, the amount was insufficient to substantially affect the compressive load-bearing system; and (2) at 0.5% fiber content, excessive fibers reduced paste fluidity and created more internal pores, compromising the geopolymer’s load-bearing capacity. Notably, when fiber contents were 0.2%, 0.3%, and 0.4%, the basalt fibers significantly improved the compressive performance of LRS geopolymers. Compared to the non-fiber-reinforced specimens, these compositions demonstrated strength enhancements of 46.6%, 60.1%, and 32.6%, respectively.

**Figure 20:**
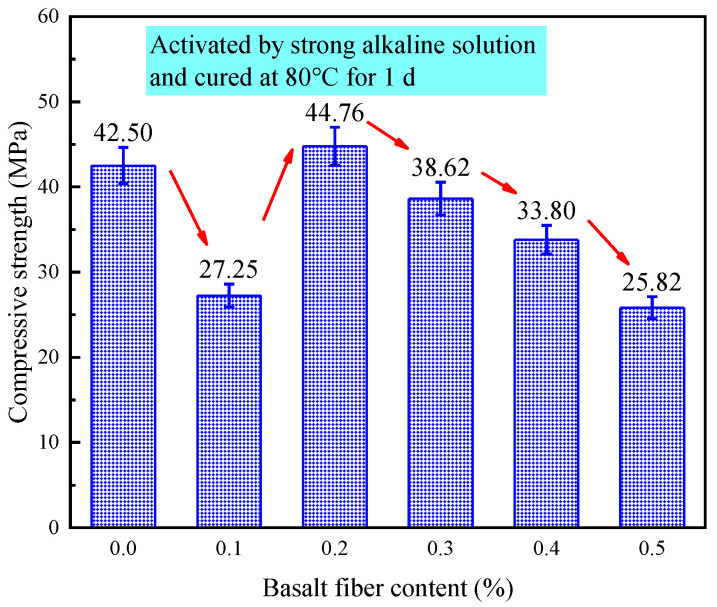
Compressive strength of LRS geopolymers with different fiber contents under strong alkali activation and cured at 80 °C for 1 d.

Figure 20 presents the effect of different fiber contents on the compressive strength of LRS lunar regolith geopolymers cured for 1 d under a strong alkaline environment at 80 °C, with compressive strengths ranging from 20.82 to 44.76 MPa. Contrary to expectations, the incorporation of basalt fibers did not lead to an overall improvement in compressive strength. Only at 0.2% fiber content was a modest, approximately 5% strength increase observed compared to the control group. The compressive strength of LRS geopolymers exhibited a distinct trend of significant reduction, slight improvement, and uniform decrease with increasing fiber content. Previous studies have demonstrated that fiber addition cannot significantly enhance the compressive strength of geopolymers [12], though the bridging effect of fibers across cracks can improve tensile properties. This finding aligns with Wang et al.’s research [34], which reported approximately 5% reduction in concrete compressive strength after adding 0.1% basalt fibers. The reduction in compressive strength of samples containing 0.1% basalt fibers may be attributed to the low fiber content at this dosage, resulting in fewer fibers to distribute the load and potentially greater susceptibility to interfacial degradation under alkaline conditions, ultimately leading to strength reduction.

**Figure 21:**
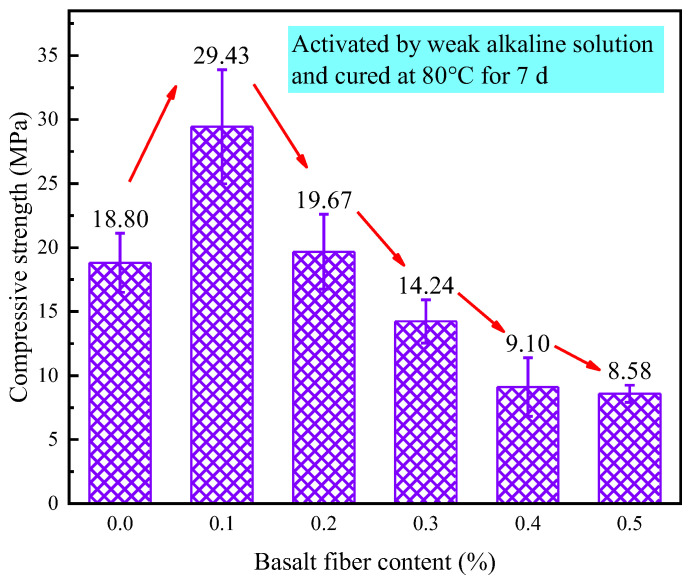
Compressive strength of LRS geopolymers with different fiber contents under weak alkali activation and cured at 80 °C for 7 d.

Figure 21 illustrates the effect of different fiber contents on the compressive strength of LRS geopolymers cured for 7 d in a weak alkaline solution at 80 °C. The compressive strength of basalt fiber-reinforced LRS geopolymers exhibited an initial sharp increase followed by a continuous decrease with increasing fiber content, with the rate of decline gradually leveling off at higher fiber contents. When the basalt fiber content was 0.1% and 0.2%, the fiber incorporation enhanced the compressive performance of LRS geopolymers, demonstrating strength increases of 56% and 5%, respectively, compared to the control group. Conversely, at higher fiber contents of 0.3%, 0.4%, and 0.5%, the compressive strength decreased by 24%, 51%, and 54%, respectively. Under weak alkaline solution with 80 °C thermal curing conditions, basalt fibers at optimal contents enhanced the compressive performance of geopolymers, while other content groups exhibited weakening effects. These findings align with research results obtained by Jalasutram et al. [33], confirming that excessive basalt fiber incorporation does not improve geopolymer compressive strength and may potentially produce negative effects.

### 4.4. Flexural Strength Under Static Loading

**Figure 22:**
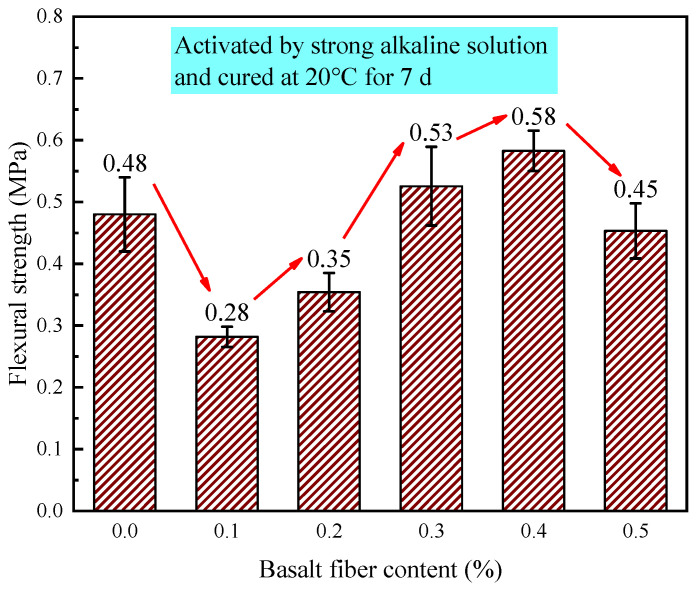
Flexural strength of LRS geopolymers with different fiber contents under strong alkali activation and cured at 20 °C for 7 d.

Figure 22 shows the effect of fiber content on the flexural strength of LRS geopolymers under strong alkaline conditions after 7 d of standard curing. Basalt fibers showed limited flexural improvement compared to their compressive strength effects. With 0.1% and 0.2% fibers, flexural strength decreased by 41.6% and 27.0%, respectively, versus the control. However, at 0.3% and 0.4% contents, strength increased by 10.4% and 20.8%, respectively. The 0.5% fiber content caused strength to decrease again below control levels.

Flexural strength followed a characteristic decreasing-increasing-decreasing trend with fiber content. Lower contents (0.1–0.2%) reduced strength by up to 41.6% due to insufficient fibers for effective load-sharing while introducing matrix porosity. Optimal reinforcement occurred at 0.3–0.4% where fibers bridged microcracks, improving strength by 10.4–20.8%. Beyond 0.5%, excessive fibers impaired workability, increasing voids and weakening microstructure.

This non-monotonic relationship aligns with findings in fiber-reinforced cementitious composites, where both insufficient and excessive fibers degrade performance. The results demonstrate the importance of optimizing fiber content for balanced mechanical properties in geopolymers.

**Figure 23:**
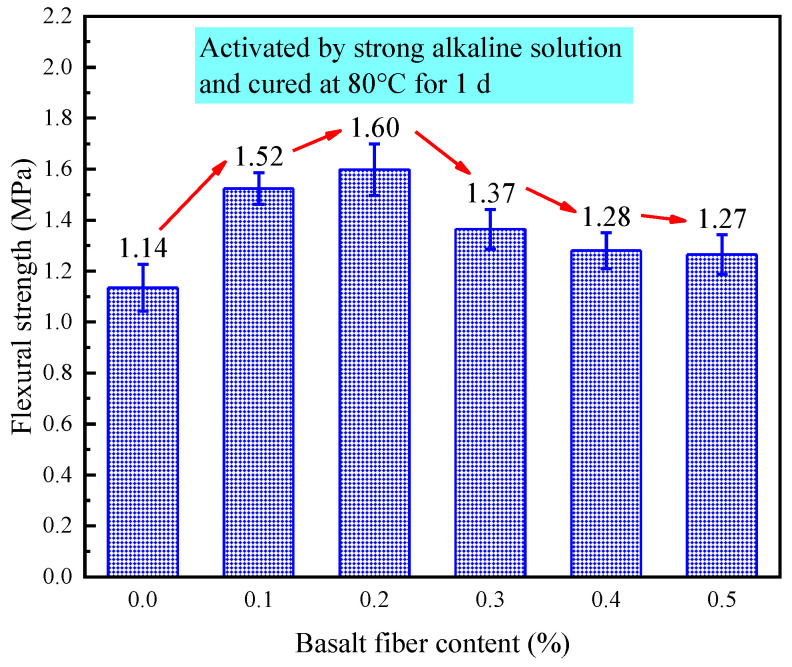
Flexural strength of LRS geopolymers with different fiber contents under strong alkali activation and cured at 80 °C for 1 d.

Figure 23 presents the effect of different fiber contents on the flexural strength of LRS geopolymers cured for 1 d under a strong alkaline environment at 80 °C. Compared to the control group, fiber incorporation enhanced the flexural strength by 11–40%. Specifically, the addition of 0.1%, 0.2%, 0.3%, 0.4%, and 0.5% basalt fibers increased the flexural strength of basalt fiber-reinforced LRS geopolymers by 33%, 40%, 20%, 12%, and 11%, respectively. The flexural strength initially increased and then decreased with increasing fiber content, reaching a peak value of 1.6 MPa at 0.2% fiber content. The most significant strength improvement occurred in the 0.0–0.1% fiber content range, followed by a decrease in the 0.2–0.3% range, after which the rate of strength reduction gradually stabilized. This behavior can be attributed to the fiber bridging effect, where the basalt fiber-reinforced LRS geopolymers continued to resist tensile stresses in the tension zone until failure, thereby improving the flexural strength—a finding consistent with the experimental results of this study [12].

**Figure 24:**
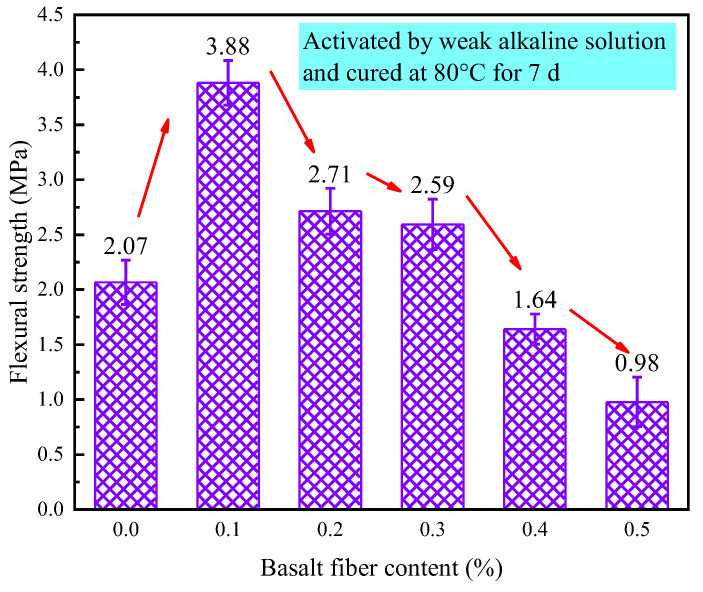
Flexural strength of LRS geopolymers with different fiber contents under weak alkali activation and cured at 20 °C for 7 d.

Figure 24 illustrates the effect of different fiber contents on the flexural strength of LRS geopolymers under a weak alkaline environment with 7 d of thermal curing at 80 °C. Compared to the control group, the basalt fiber-reinforced LRS geopolymers exhibited significant strength improvements at fiber contents of 0.1%, 0.2%, and 0.3%, with enhancement rates of 87%, 31%, and 20%, respectively. However, when fiber contents reached 0.4% and 0.5%, the flexural strength decreased by 21% and 53% compared to the control. The flexural strength demonstrated an initial increase followed by a decrease with increasing basalt fiber content. At optimal fiber contents of 0.2% and 0.3%, the flexural strength stabilized at levels significantly higher than those of the control group. Notably, the most dramatic improvement occurred at 0.1% fiber content, where the flexural strength increased sharply by 87%, clearly demonstrating that an appropriate amount of basalt fibers can substantially enhance the flexural performance of geopolymers.

The experimental study conducted in a strong alkaline solution with 80 °C thermal curing showed that the flexural strength remained superior to that of the control group even with excessive fiber incorporation. However, under weak alkaline high-temperature conditions, excessive fiber content adversely affected the flexural performance of LRS geopolymer. The primary reasons were as follows: (1) excessive fiber content increased internal porosity in the specimens; (2) in weak alkaline environment, overabundance of basalt fibers reduced the pH value of the paste, decreasing alkalinity and consequently reducing the solubility of geopolymeric particles, leading to flexural strength degradation [32]; (3) the weak alkaline solution exhibited poorer fluidity than strong alkaline solution, and excessive fiber incorporation further reduced this fluidity, resulting in additional weakening of flexural strength.

### 4.5. Elastic Modulus Under Static Loading

**Figure 25:**
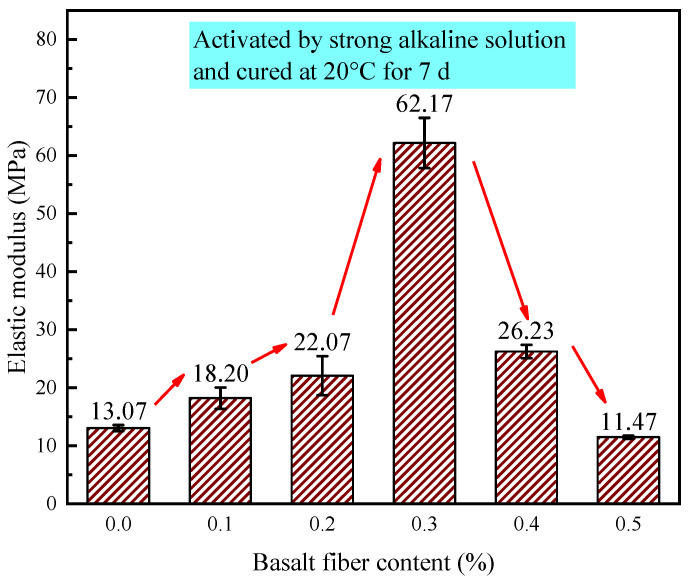
Elastic modulus of LRS geopolymers with different fiber contents under strong alkali activation and cured at 20 °C for 7 d.

In this study, the load-displacement curve obtained from the actuator displacement data was used to calculate the elastic modulus of the material. This method, which involves precise displacement control and real-time load acquisition, accurately captures the stress-strain response of the material in the elastic stage, offering high measurement accuracy and reliability. Figure 25 shows the elastic modulus of LRS geopolymers after 7 d of curing in a strong alkaline solution under standard conditions. In this study, the ASTM C469 standard was adopted [35]. Values ranged from 11.47 to 62.17 MPa, peaking at 0.3% fiber content with a 3.7-fold increase over the control. The modulus initially rose, then declined with increasing fiber content, with optimal performance occurring at 0.2–0.4% fiber content. All fiber-reinforced specimens except the 0.5% group showed significantly higher modulus than the control, demonstrating that appropriate basalt fiber content (0.1–0.4%) effectively enhances geopolymer stiffness. These results provide valuable guidance for optimizing mechanical performance in fiber-reinforced geopolymers.

**Figure 26:**
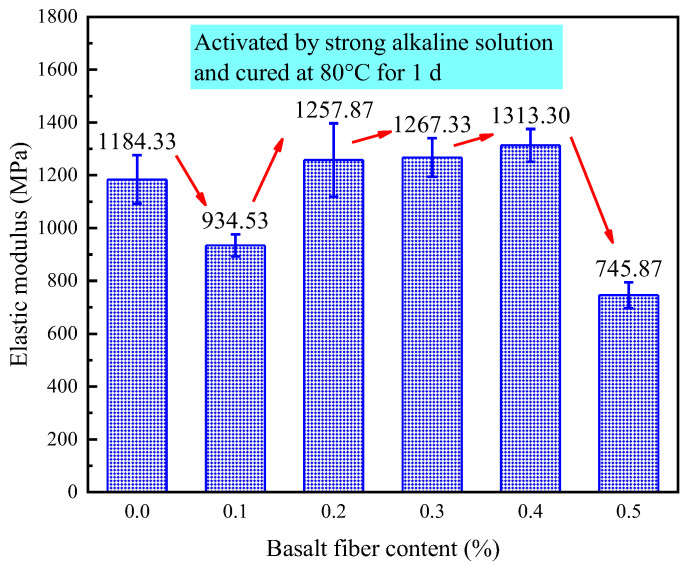
Elastic modulus of LRS geopolymers with different fiber contents under strong alkali activation and cured at 80 °C for 1 d.

Figure 26 presents the elastic modulus of LRS geopolymers after 1 d of curing in a strong alkaline solution at 80 °C, with values ranging from 745.87 to 1313.3 MPa. The modulus exhibited a three-stage response to fiber content: initial reduction at 0.1% fiber content, significant increase at 0.2–0.4% fiber content, and sharp decline at 0.5% fiber content. Optimal fiber contents of 0.2%, 0.3% and 0.4% increased modulus by 6.2%, 7.0% and 10.9%, respectively, while 0.1% and 0.5% contents decreased it. This demonstrates that moderate fiber additions between 0.2–0.3% enhance stiffness, while insufficient amounts below 0.1% or excessive amounts above 0.4% reduce performance. The high modulus values indicate rapid early strength development under these curing conditions, though with reduced deformation capacity. These results provide valuable insights for optimizing early-age mechanical properties in fiber-reinforced geopolymers.

**Figure 27:**
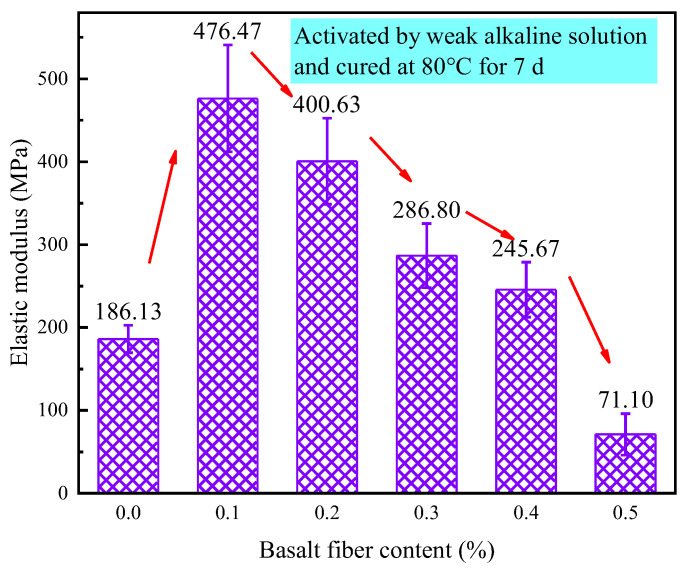
Elastic modulus of LRS geopolymers with different fiber contents under weak alkali activation and cured at 80 °C for 7 d.

Figure 27 shows the elastic modulus variation of LRS geopolymers after 7 d of curing in a weak alkaline solution at 80 °C. Values ranged from 71.1 to 476.47 MPa, initially rising, then declining with fiber content. Compared to control, 0.1%, 0.2%, 0.3% and 0.4% fiber contents increased modulus by 156.0%, 115.3%, 54.1% and 32.0%, respectively, while 0.5% decreased it by 61.7%. This shows moderate fiber additions (0.1–0.3%) significantly enhance stiffness under these conditions, but excess fibers (>0.4%) cause degradation. Higher fiber contents reduced stiffness but slightly improved ductility, suggesting potential for balancing these properties in material design.

### 4.6. Ultimate Strain Under Static Loading

**Figure 28:**
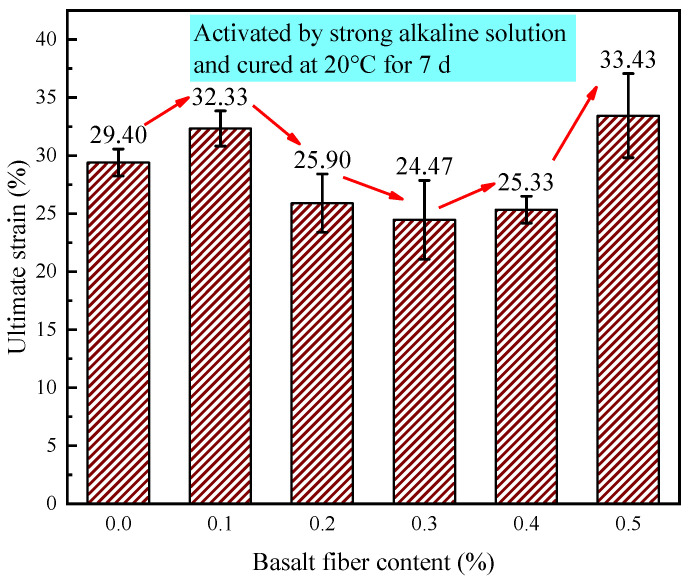
Ultimate strain of LRS geopolymers with different fiber contents under strong alkali activation and cured at 20 °C for 7 d.

Figure 28 shows how fiber content affects the ultimate strain of LRS geopolymers after 7 d of standard curing (20 °C) in strong alkaline conditions. Ultimate strain values ranged from 20.3% to 33.43%, showing nonlinear variation. The 0.1% and 0.5% fiber groups increased strain by 2.93% and 4.03%, respectively, versus the control, while the 0.2–0.4% groups decreased by 1.2–2.8%. This occurred because the geopolymer matrix already had good ductility under these curing conditions, limiting fiber reinforcement potential. At 0.1% content, fibers enhanced ductility without excessive porosity, while at 0.5%, abundant fibers compensated for increased porosity through effective crack bridging. However, intermediate contents (0.2–0.4%) created porosity that outweighed fiber benefits, reducing deformation capacity and ultimate strain.

**Figure 29:**
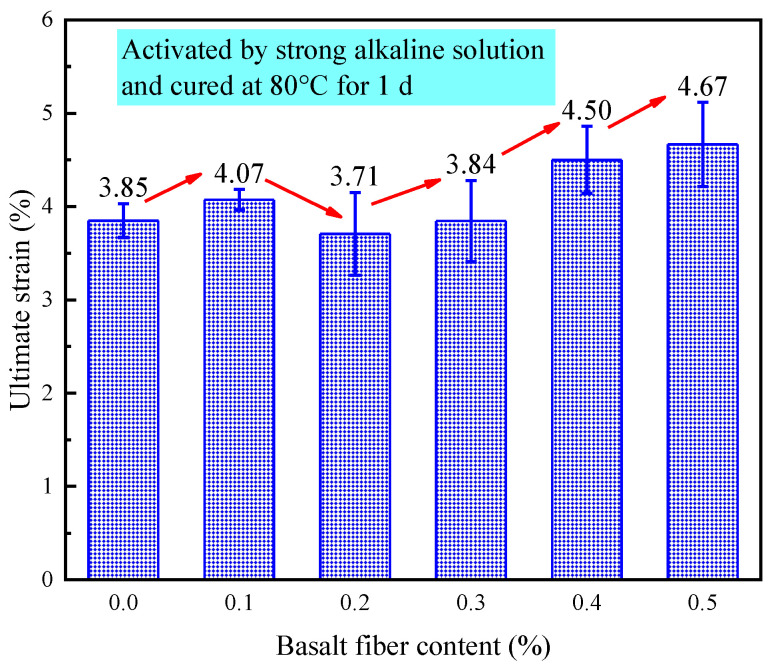
Ultimate strain of LRS geopolymers with different fiber contents under strong alkali activation and cured at 80 °C for 1 d.

Figure 29 demonstrated the effect of varying fiber contents on the ultimate strain of LRS geopolymers subjected to 1 d of curing at 80 °C in a strong alkaline environment. Experimental results revealed that all specimens exhibited relatively low ultimate strain values ranging from 3.85% to 4.67%, confirming the material’s brittle failure characteristics under these conditions. The fiber content showed a non-monotonic influence on ultimate strain performance: while 0.1% fiber content increased ultimate strain by 5.7%, 0.2% and 0.3% contents resulted in reductions of 3.6% and 0.26%, respectively. However, higher fiber contents of 0.4% and 0.5% produced significant enhancements of 16.9% and 21.3%. Importantly, although the absolute differences in ultimate strain remained limited, the incorporation of optimal fiber amounts (0.3–0.4%) led to substantial improvements in both compressive and flexural strengths. This behavior indicates that the fiber-reinforced geopolymers retained partial load-bearing capacity even after matrix failure, clearly demonstrating the fiber toughening effect.

**Figure 30:**
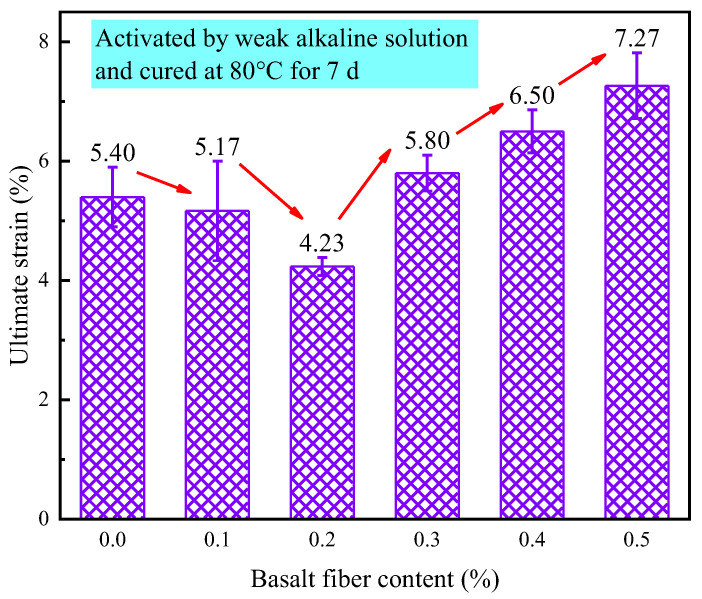
Ultimate strain of LRS geopolymers with different fiber contents under weak alkali activation and cured at 80 °C for 7 d.

Figure 30 shows how fiber content affects the ultimate strain of LRS geopolymers after 7 d of curing in a weak alkaline solution at 80 °C. Ultimate strain varied nonlinearly between 4.23% and 7.27%, initially decreasing, then increasing with fiber content. Specimens with 0.1% and 0.2% fibers showed 4.3% and 21.7% reductions versus control, while 0.3%, 0.4% and 0.5% fibers increased strain by 7.4%, 20.4% and 34.6%. This demonstrates that basalt fibers above 0.3% effectively enhance deformation capacity under these conditions. The nonlinear behavior arises from varying fiber-matrix bonding: contents ≤0.2% provide insufficient fiber distribution to constrain the matrix, while ≥0.3% forms a continuous network enabling efficient stress transfer and improved ductility.

## 5. Discussions

**Figure 31:**
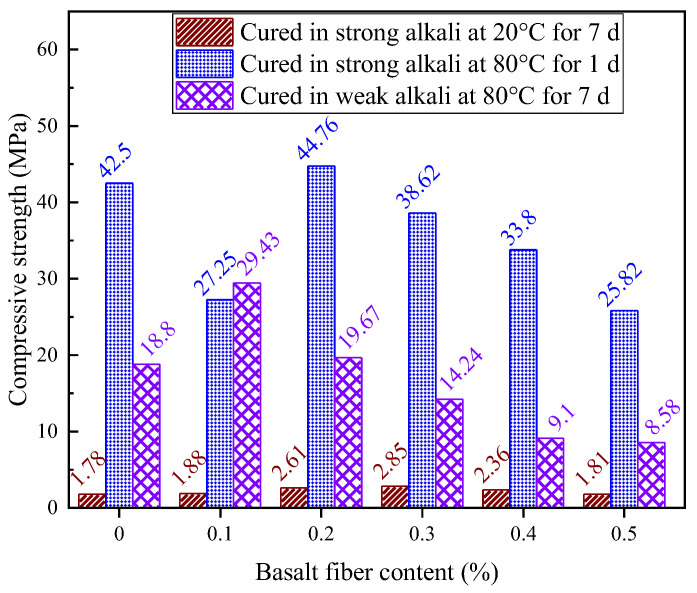
Comparison of compressive strength of LRS geopolymers in different environments.

Figure 31 compares the compressive strength of LRS geopolymers under varying alkali-activation and curing conditions. Basalt fiber incorporation significantly enhanced compressive strength compared to the control group, with the optimal improvement (56.5%) achieved at 0.1% fiber content in a weak alkaline environment (7 d, 80 °C). However, insufficient or excessive fiber content degraded the load-bearing structure, reducing strength.

Strong alkali-activated specimens cured at 80 °C for 1 d exhibited the highest compressive strength (44.76 MPa), while weak alkali-activated samples under the same curing conditions showed ~50% lower strength. Standard-cured, strong alkali specimens performed the worst. Curing temperature was the dominant strength factor, with 80 °C curing accelerating early-age strength development. Alkali type also played a critical role: strong alkali solutions improved early strength but caused fiber corrosion at high temperatures, increasing porosity and weakening reinforcement. Weak alkali systems with optimal fiber content provided balanced compressive-flexural performance. Future work will focus on weak alkali systems to refine fiber reinforcement mechanisms and mix design.

**Figure 32:**
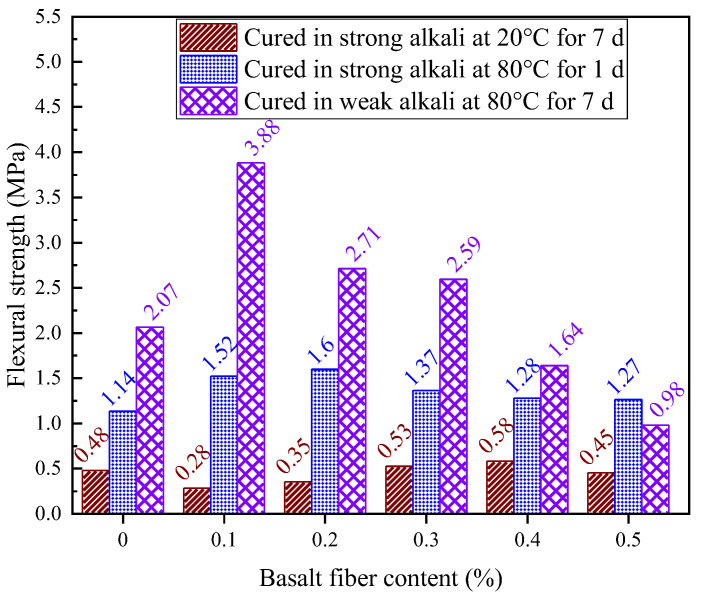
Comparison of flexural strength of LRS geopolymers in different environments.

Figure 32 shows that flexural strength optimization occurred in weak alkaline environments with 7 d/80 °C curing, where 0.1% basalt fiber content increased strength by 87.1% (3.88 MPa). Specimens exhibited a characteristic strength peak at this fiber content, with greater performance variation than strong alkaline samples.

While strong alkali activation (1 d/80 °C) enhanced compressive strength through improved particle solubility, it simultaneously corroded fibers at high temperatures. The resulting porosity reduced flexural performance by limiting ductility and fiber toughening effects. Weak alkali activation with 7 d/80 °C curing and 0.1% fibers provided an optimal balance, maintaining compressive strength while maximizing flexural improvement (87.1%), demonstrating superior reinforcement without alkali corrosion.

**Figure 33:**
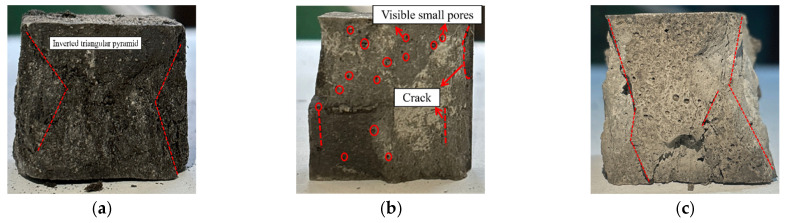
Comparison of compressive failure modes of LRS geopolymer compressive test blocks in different environments. (**a**) Curing in strong alkali at 20 °C for 7 d. (**b**) Strong alkali curing at 80 °C for 1 d. (**c**) Weak alkali curing at 80 °C for 7 d.

Figure 33 compares failure modes of geopolymer specimens under different curing conditions. Standard-cured samples showed progressive flattening and ductile failure, while 80 °C-cured specimens exhibited brittle fracture with whitish surfaces and spalling. Strong alkali activation at 80 °C caused irregular failure patterns due to fiber corrosion-induced porosity. In weak alkali/80 °C systems, unreinforced specimens failed via hoop effect, whereas fiber-reinforced ones developed bulged cross-sections with extensive cracking, demonstrating fiber-controlled fracture propagation.

**Figure 34:**
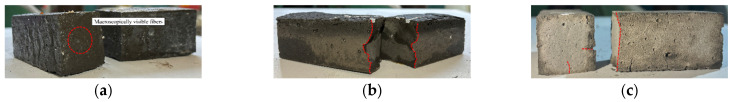
Comparison of flexural failure modes of LRS geopolymer compressive test blocks in different environments. (**a**) Curing in strong alkali at 20 °C for 7 d. (**b**) Strong alkali curing at 80 °C for 1 d. (**c**) Weak alkali curing at 80 °C for 7 d.

Figure 34 compares failure modes of geopolymer flexural specimens under different curing conditions. Standard-cured specimens exhibited darker surfaces, softer texture, and ductile failure, though with lower flexural strength. In contrast, 80 °C-cured specimens showed progressively lighter whitish surfaces and brittle failure behavior.

Basalt fiber incorporation led to wider crack patterns, demonstrating effective crack-bridging and ductility enhancement. Weak alkali-activated specimens with fibers displayed distinct whitish core discoloration, attributed to white gel-like polymer formation from the fiber-LRS-weak alkali interaction under high temperatures. Optimal fiber content improved both mechanical properties and matrix bonding strength in this system.

Figure 9, Figure 10 and Figure 11 illustrate the curing-condition-dependent stress-strain behaviors of the geopolymers. Standard-cured specimens demonstrated 5–6 times greater deformation capacity but only 10% of the compressive strength compared to their 80 °C-cured counterparts. The strong alkali/1 d −80 °C system achieved maximum strength with strain-hardening behavior, except for the 0.5% fiber content, which exhibited linear-elastic characteristics. Optimal fiber addition transformed the strong alkali/standard-cured curves from linear-elastic to strain-hardening due to increased elastic modulus. Weak alkali/7 d −80 °C specimens showed fiber-dependent transitions (linear-elastic → strain-hardening → linear-elastic), confirming that moderate fiber content optimizes mechanical performance, while excessive or insufficient fiber incorporation diminishes it.

**Figure 35:**
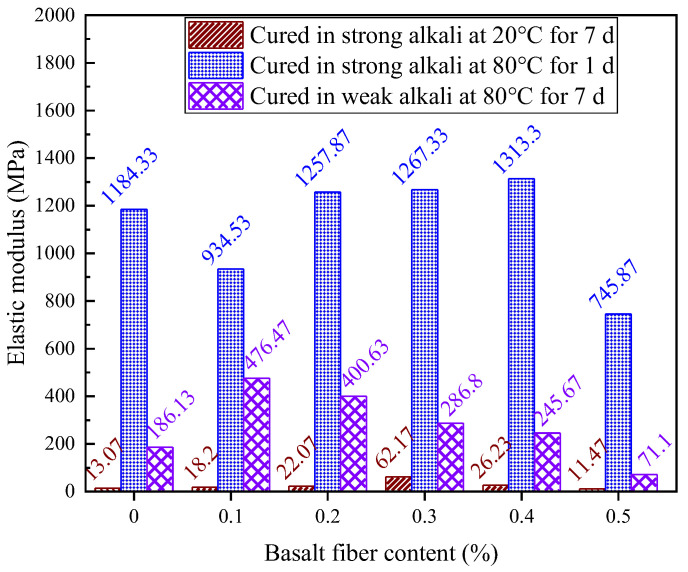
Comparison of the elastic modulus of LRS geopolymers in different environments.

As illustrated in Figure 35, the curing temperature and alkali solution type critically control geopolymer elastic modulus, with strong alkali/80 °C curing achieving the highest values. The fiber reinforcement effect exhibited strong environmental dependence: 0.3% fibers maximized modulus enhancement in strong alkali/20 °C systems, while only 0.1% fibers yielded significant gains in weak alkali/80 °C conditions, and 0.4% fibers provided marginal improvement in strong alkali/80 °C systems. Each condition displayed a distinct optimal fiber content range, with deviations reducing performance, establishing practical guidelines for engineering geopolymer composites with tailored mechanical properties.

**Figure 36:**
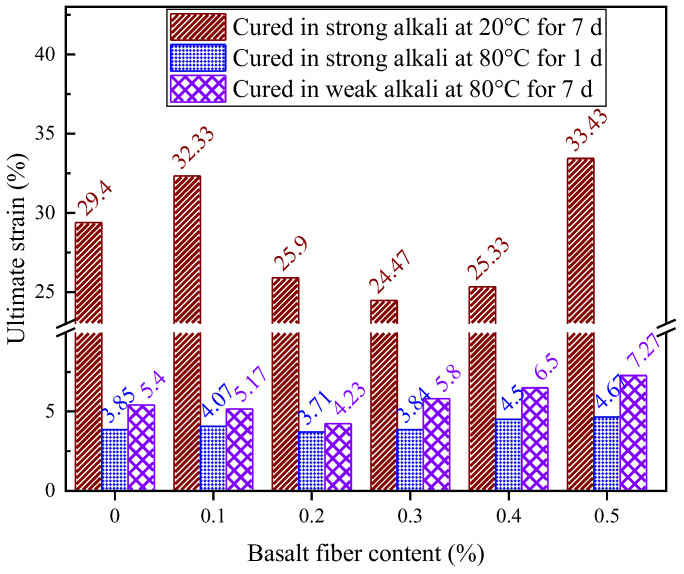
Comparison of ultimate strains of LRS geopolymers in different environments.

Figure 36 reveals that the ultimate strain in geopolymers was significantly affected by curing conditions. Standard-cured (20 °C/7 d) strong alkali specimens exhibited the highest ultimate strain, while strong alkali/80 °C/1 d samples showed the lowest values, indicating reduced deformation capacity under high-temperature curing. Weak alkali systems at 80 °C demonstrated superior strain performance compared to strong alkali equivalents. Basalt fiber reinforcement improved ultimate strain at optimal contents, though excessive fibers caused mechanical property trade-offs, emphasizing the importance of balanced fiber addition.

The 0.3% basalt fiber-reinforced LRS geopolymer under strong alkali conditions (20 °C/7 d of curing, Table 6) exhibited optimal balanced performance, demonstrating a 60.1% compressive strength increase over the control while maintaining near-peak flexural strength (just 0.05 MPa below maximum). This formulation showed significantly enhanced stiffness (3.7 × higher elastic modulus) with characteristic “slender” stress-strain behavior compared to the control’s “flat” curve, and preserved adequate ductility despite an 11.9% strain reduction. These results indicate that 0.3% fiber content optimally modifies the microstructure to achieve a superior strength-stiffness-ductility balance.

The 0.2% basalt fiber-reinforced LRS geopolymer showed optimal performance under strong alkali conditions (80 °C/1 d of curing, Table 7), achieving concurrent 5.3% higher compressive strength, 40.4% greater flexural strength, 6.2% increased elastic modulus, and 5.7% improved ultimate strain compared to controls. The characteristic “slender” stress-strain curve with delayed peak stress demonstrated enhanced load-bearing capacity and ductility, indicating ideal microstructural modification at this fiber content for balanced mechanical properties.

The 0.1% basalt fiber-reinforced CQU-1 LRS geopolymer demonstrated optimal performance under weak alkali conditions (80 °C/7 d of curing, Table 8), exhibiting remarkable enhancements including 56.5% higher compressive strength, 87.4% greater flexural strength, and 155.5% increased elastic modulus while maintaining comparable ductility (only 4.3% strain reduction). The characteristic “slender” stress-strain curve with delayed peak stress indicated superior load-bearing capacity and balanced mechanical properties, suggesting ideal fiber-matrix interaction at this content for the CQU-1 system.

## 6. Conclusions

This study systematically investigated the influence mechanisms of basalt fiber content on the mechanical properties of LRS geopolymers under different curing conditions. Through a comprehensive experimental design incorporating multiple variables—including alkali solution type (strong/weak alkaline), curing temperature (20 °C/80 °C), and curing duration (1 d/7 d)—we thoroughly analyzed key mechanical indicators such as stress-strain characteristics, failure modes, compressive strength, flexural strength, elastic modulus, and ultimate strain. The main research conclusions are as follows:

(1) Curing conditions were found to be the decisive factor affecting geopolymer performance. Specimens cured in a strong alkaline environment at 20 °C for 1 d failed to achieve proper hardening. However, when the curing duration was extended to 7 d with 0.3% fiber content, the optimal comprehensive performance was obtained (compressive strength: 2.85 MPa, flexural strength: 0.53 MPa), exhibiting ductile failure characteristics and “flat”-shaped stress-strain curves.
(2) High-temperature curing at 80 °C significantly improved material properties. Under a strong alkaline environment with 1 d of curing at 80 °C, specimens containing 0.2% basalt fibers demonstrated the highest compressive strength (44.76 MPa) and flexural strength (1.60 MPa), but showed brittle failure characteristics and “slender”-shaped stress-strain curves.
(3) A weak alkaline environment exhibited unique advantages. With 7 d of curing at 80 °C and 0.1% fiber content, the specimens not only reached peak flexural strength (3.88 MPa) but also displayed optimal stress-strain curve morphology, indicating that a weak alkaline environment is more conducive to achieving synergistic effects between fibers and matrix.
(4) Temperature effect analysis revealed that standard curing promotes ductility development (ultimate strain: 24.47%), while 80 °C high-temperature curing significantly enhances stiffness (maximum elastic modulus: 1207.87 MPa) and strength properties at the expense of reduced material ductility. Comparison of alkali solutions showed that although a strong alkaline solution provides more pronounced activation effects, it causes fiber corrosion under high temperatures, leading to increased porosity, whereas a weak alkaline solution facilitates the formation of more stable fiber-matrix interface structures. Strength relationship analysis confirmed that the flexural strength of geopolymers is approximately 1/6–1/10 of the compressive strength, providing important reference values for engineering applications.

## Figures and Tables

**Table 1:** Comparison of the chemical composition of lunar regolith simulant (%).

Sample	SiO_2_	TiO_2_	Al_2_O_3_	Fe_2_O_3_	CaO	MgO	Na_2_O	K_2_O	P_2_O_5_
CQU-1	45.31	2.80	15.01	15.67	8.34	3.41	4.50	3.33	0.65
HUST-1 [9]	48.23	2.96	18.29	11.19	7.89	4.41	3.70	2.15	0.50
BH-1 [10]	43.30	2.90	16.50	16.70	8.80	3.00	3.80	3.30	0.70
CAS-1 [8]	49.24	1.91	15.80	11.47	7.20	8.72	3.08	1.03	0.30
CE-5 [27]	42.20	5.00	10.80	22.50	11.00	6.48	0.26	0.19	0.23
Apollo 14 [28]	48.10	1.70	17.40	10.40	10.70	9.40	0.70	0.55	0.51

**Table 2:** Parameters of sodium silicate solution.

SiO_2_/wt.%	Na_2_O/wt.%	Modulus	Density (20 °C) g/mL	Baumé Degree (20 °C)	Transparency/%	Fe/%
26.5	8.3	3.3	1.368	39.0	85.0	0.005

**Table 3:** Physical properties of basalt fibers.

Monofilament Diameter	Density (g/cm^3^)	Elastic Modulus (GPa)	Tensile Strength (MPa)	Service Temperature	Bonding Temperature
10 μm	2.63~2.65	91~110	3000~4800	−269–650 °C	1050 °C

**Table 4:** Alkali solution parameters.

Type of Solution	Modulus	Alkali Content	Water-Binder
Strong alkali	1.5	10	0.25
Weak alkali	3.3	8.3	0.456

**Table 5:** Mix proportion.

Number	Water/ Binder Ratio	Na_2_O Content (%)	Modulus	LRS (g)	NaOH (g)	Na_2_SiO_3_ (g)
Strong alkaline solution	0.25	10	1.5	100	7.05	54.67
Weak alkaline solution	0.456	8.3	3.3	100	0	100

**Table 6:** Comparison of mechanical properties between the optimal content group and the control group after 7 d of curing at 20 °C.

Number	Compressive Strength (MPa)	Flexural Strength (MPa)	Elastic Modulus (MPa)	Ultimate Strain (%)
Treatment group	1.78	0.48	13.07	29.4
Optimal content (0.3%)	2.85	0.53	62.17	24.5

**Table 7:** Comparison of the mechanical properties of the optimal content group and the control group after 1 d of curing at 80 °C.

Number	Compressive Strength (MPa)	Flexural Strength (MPa)	Elastic Modulus (MPa)	Ultimate Strain (%)
Treatment group	42.5	1.14	1184.33	3.85
Optimal content (0.2%)	44.76	1.6	1207.87	3.71

**Table 8:** Comparison of the mechanical properties of the optimal content group and the control group after 7 d of curing at 80 °C.

Number	Compressive Strength (MPa)	Flexural Strength (MPa)	Elastic Modulus (MPa)	Ultimate Strain (%)
Treatment group	29.43	2.07	186.13	5.4
Optimal content (0.1%)	18.8	3.88	476.47	5.17

## Data Availability

The original contributions presented in the study are included in the article; further inquiries can be directed to the corresponding author.
